# Formation of charged ferroelectric domain walls with controlled periodicity

**DOI:** 10.1038/srep15819

**Published:** 2015-10-30

**Authors:** Petr S. Bednyakov, Tomas Sluka, Alexander K. Tagantsev, Dragan Damjanovic, Nava Setter

**Affiliations:** 1 Ceramics Laboratory, EPFL – Swiss Federal Institute of Technology, 1015 Lausanne, Switzerland

## Abstract

Charged domain walls in proper ferroelectrics were shown recently to possess metallic-like conductivity. Unlike conventional heterointerfaces, these walls can be displaced inside a dielectric by an electric field, which is of interest for future electronic circuitry. In addition, theory predicts that charged domain walls may influence the electromechanical response of ferroelectrics, with strong enhancement upon increased charged domain wall density. The existence of charged domain walls in proper ferroelectrics is disfavoured by their high formation energy and methods of their preparation in predefined patterns are unknown. Here we develop the theoretical background for the formation of charged domain walls in proper ferroelectrics using energy considerations and outline favourable conditions for their engineering. We experimentally demonstrate, in BaTiO_3_ single crystals the controlled build-up of high density charged domain wall patterns, down to a spacing of 7 μm with a predominant mixed electronic and ionic screening scenario, hinting to a possible exploitation of charged domain walls in agile electronics and sensing devices.

Certain interfaces between two distinct materials like GaAs/Al_x_Ga_1−x_As or LaAlO_3_/SrTiO_3_ carry a high mobility two-dimensional electron or hole gas (2DG) and are widely exploited e.g. in high frequency field effect transistors. These 2DGs are confined to fixed heterogeneous interfaces^1^,^2^ or surfaces^3^,^4^. Recently, we showed experimentally^5^ a new type of quasi-2DG, which is not confined to any fixed interface but to a movable and compositionally homogeneous ferroelectric domain wall. The quasi-2DG can be created, displaced and erased electrically inside a crystal monolith of the widely used low conductive proper ferroelectric BaTiO_3_ and, hence, such a wall can be potentially applied as a rewritable mobile conducting channel inside a working device. This effect can function also in other ferroelectrics promising a new degree of freedom in electronic design. However, the current state-of-the-art critically lacks even basic engineering know-how that would allow quasi-2DG formation and manipulation.

Ferroelectric materials consist of domains that are spontaneously polarised in one of the symmetry-permitted directions of the material. Each two adjacent domains are separated by compositionally homogeneous interface having a typical width of 1–10 nm. These interfaces usually meet the condition of electrostatic compatibility, i.e. the normal component of the spontaneous polarisation is continuous across the interface^6^. They carry no net bound charge and are hence called neutral domain walls (NDW). Alternatively, charged domain walls (CDW) may exist, which carry bound charge due to a jump of this polarisation component at the wall, violating the condition of electrostatic compatibility. Figure 1 illustrates simple examples of 180° NDW (Fig. 1a) and CDWs (Fig. 1b–d) between domains with antiparallel polarisation orientation.

Of a limited experimental interest in the past^7^,^8^,^9^,^10^, the occurrence of CDWs have been recently documented experimentally in classical proper^5^,^11^,^12^,^13^,^14^, improper^15^ and hybrid improper ferroelectrics^16^, and in organic ferroelectrics^17^. The interest in CDWs is primarily motivated by the phenomenon, which was theoretically predicted some 40 years ago^18^. It was only recently experimentally confirmed^5^ that CDWs can carry a degenerate highly-conductive electron gas though some indications were reported earlier^19^. Potentially promising as well is the theoretical prediction of enhanced piezoelectric response in ferroelectrics as a function of increased CDWs density^20^. Methods to intentionally create CDWs are, however, still obscure.

Here we present the theory and methods of controlled formation of CDW patterns. First, we propose classification and relevant terminology for the phenomenon. Then we outline the theoretical background for the formation of CDWs, using mainly energy considerations, which identifies the favourable conditions for CDW formation. Finally, we apply the theory to experiments where we demonstrate the controlled formation of CDW patterns with decreasing domain width down to 7 μm. All domain patterns with CDWs were fabricated in BaTiO_3_ crystals obtained from the same supplier as ones studied in^5^, where it was shown that the obtained domain boundaries are highly conductive interfaces.

## Results

### Classification of Charged Domain Walls

The violation of the electrostatic compatibility at CDWs leads to a nonzero net divergence of spontaneous polarisation 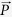 and the appearance of a *bound charge* density 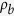 at the wall as it follows from


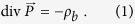


**Figure 1:**
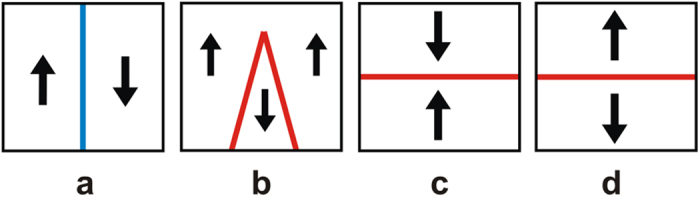
Neutral and charged 180° domain walls (schematic illustration). (**a**) Neutral domain wall which fully satisfies the condition of electrostatic compatibility, i.e. the normal component of the spontaneous polarisation (indicated with arrows) is continuous across the wall. (**b**) Domain configuration containing domain walls that are partly charged due to their slight inclination with respect to the polarisation direction; such configurations inevitably appear during polarisation reversal starting from a single-domain state. (**c**,**d**) charged domain walls, where the condition of electrostatic compatibility is strongly violated, i.e. the jump of the normal component of the spontaneous polarisation comparable to the polarisation itself. The head-to-head configuration (**c**) carries positive bound charge and the tail-to-tail configuration (**b**,**d**) negative bound charge.

Depending on the sign of the charge, one calls the CDWs either head-to-head (H-H) oriented if the charge is positive (Fig. 1c) or tail-to-tail (T-T) oriented if the charge is negative (Fig. 1b,d).

The bound charge 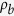 at CDWs produces an electric field, which penalises CDW formation with increased electrostatic energy. However, the main penalty comes from the fact that the field always has a depolarising effect on the adjacent domains as it is always directed (at least partly) against the polarisation. If this field exceeds the thermodynamic coercive limit of the material, ferroelectricity in the adjacent domains is destabilised and the CDW must vanish. The depolarisation field can be significantly reduced below the critical limit when the bound charge is compensated either by mobile defects or free electrons or holes.

Therefore, CDWs can be categorised into two types: (i) Weakly charged domain walls (wCDW) corresponding to the case where the depolarisation field created by the bound charge (dielectric approximation, i.e. in the absence of screening charges) is not large enough to destabilise ferroelectricity in the adjacent domains and (ii) strongly charged walls (sCDW) corresponding to the case where the bound charge at the CDW is sufficient to create a depolarisation field which, in the dielectric approximation, destabilises ferroelectricity in the adjacent domains. In fact, sCDWs in common ferroelectrics require almost perfect compensation in order not to be destabilised.

WCDWs form at least temporarily during each 180° polarisation switching because domain walls of a nucleus formed upon switching are inevitably inclined with respect to the polarisation and therefore partly charged (Fig. 1b). The wCDW misalignment from neutral configuration during switching is, however, small and its resulting depolarisation field is not sufficient to destabilise ferroelectricity. Nevertheless, the bound charge at wCDWs can still induce specific DW properties. For instance, elevated conduction of the wall was documented in^12^,^21^.

**Figure 2:**
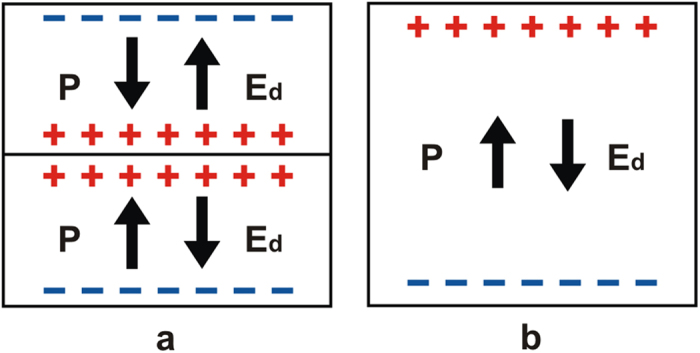
Bound charges and depolarisation fields in the dielectric approximation. (**a**) Sample with a head-to-head charged domain wall. **(b**) Single domain ferroelectric plate. Directions of the polarisation *P* and depolarisation field *E*_d_ are indicated with arrows. “ + ” and “−” indicate positive and negative bound charges. In the dielectric approximation, no free charges are present in the system.

At the CDWs schematically shown in Fig. 1c,d, the condition of electrostatic compatibility is essentially violated, i.e. the jump of the normal component of the spontaneous polarisation is comparable to the polarisation itself. In proper ferroelectrics having usual values of spontaneous polarisation (from a few to tens of 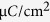), such walls should be classified as sCDW. Let us show that, indeed, in a BaTiO_3_-type material, such walls cannot exist in the dielectric approximation, i.e. without screening of the bound charge with free charge carries. The depolarisation electric field *E* created solely by the bound charges (in the absence of free charges) at the H-H sCDW shown in Fig. 2a is easily found because the electric displacement 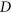 produced by the free charges vanishes. Then, 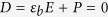 defined in terms of the ferroelectric contribution to the polarisation *P*, and the background dielectric permittivity 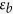 implies


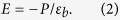


One readily checks that, in the dielectric approximation, a depolarisation field of the same size appears in a ferroelectric single-domain plate with a normal orientation of the polarisation (Fig. 2b). Thus, the problem of a destabilisation of ferroelectricity in a sample with sCDW is quantitatively identical to that of a ferroelectric plate where it was shown that such a depolarisation field leads to a drop of the Curie temperature *T*_0_ by^6^


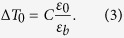


Here, 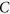 is the Curie-Weiss constant and 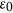 is the permittivity of vacuum. According to Eq. (3), in BaTiO_3_ (with 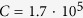 K and 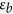/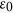 = 7)^22^, 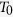 would be shifted by 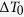 = 24000 K. Thus, the presence of the non-screened H-H configuration in question will lead to the same shift of 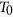. Similar estimates apply to other perovskite ferroelectrics. Such shifts of 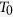 are physically impossible, implying that, in the dielectric approximation, similar to the case of ferroelectricity in a single-domain plate, such CDWs cannot exist. In practice, the ferroelectricity in a single-domain plate is possible primarily because of the *external screening* of the polarisation charge with adsorbates, i.e. with the environmental free charged species on the sample surface. Of course, in the case of electroded and short-circuited samples, screening is provided by external electronic carriers. The situation with sCDW is very different: Since the positive bound charge is located inside the insulating material (Fig. 2a), *external screening* is fundamentally impossible. Thus, the existence of sCDW requires pure *internal screening*, i.e. screening by free charges from the bulk of the material. Actually, in view of the aforementioned huge shift of 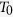, such screening must be virtually complete. In the case of electronic screening, as first indicated by Vul *et al.*^18^,^23^, a degenerate electron gas can readily appear at sCDWs. A qualitatively identical conclusion applies also to non −180° sCDWs in typical proper ferroelectrics, for example, to 90° sCDW in tetragonal BaTiO_3_, where the polarisation in the adjacent domain makes a 45° angle with the wall; such walls are comprehensively addressed further on in the paper.

Despite the essential need for very strong internal screening in nominally dielectric materials, sCDW were documented in a number of proper ferroelectrics having regular values of the the Curie-Weiss constant^5^,^7^,^10^,^11^,^24^,^25^.

Configurations with an appreciable jump of the normal component of the polarisation at the wall (like in Fig. 1c,d) are not exclusive to proper ferroelectrics. Such configurations can appear also in so-called weak ferroelectrics and improper ferroelectrics. Weak ferroelectrics^6^,^26^,^27^ are proper ferroelectrics, which exhibit very small values of their Curie-Weiss constants, in the range 3–30 K. It is clear from Eq. (3) that for such small Curie-Weiss constants, the presence of bound charge at CDWs leads merely to a small reduction of the Curie-Weiss temperature. Thus, ferroelectricity in the domains adjacent to a CDW in a weak ferroelectric is not destabilised, except for in close vicinity of the transition temperature. We therefore categorise these walls as wCDW. A similar situation occurs in improper ferroelectrics. It has been shown^6^,^28^ that the depolarisation field does not imply any shift of the ferroelectric phase transition of an improper ferroelectric, leading only to a reduction of its spontaneous polarisation. Thus, in improper ferroelectrics, the bound charge at walls with an appreciable jump of the normal component of the polarisation does not destabilise ferroelectricity in the domains. Such DWs fall also into the category of wCDWs. The existence of CDWs in improper ferroelectrics does not necessarily require screening of the bound charge, which may explain their regular existence in improper ferroelectric semiconductors like YMnO_3_ and ErMnO_3_ etc.^15^,^29^,^30^.

### Factors controlling the formation energy of charged domain walls

The occurrence of sCDWs is controlled to a great extent by the formation energy of the walls, which in turn is sensitive to the details of the screening mechanism and the availability of charge exchange between the crystal and its exterior (electrode or/and ambient atmosphere). Both, electrons (holes) and charged ionic species, can participate in the bound-charge screening at the sCDW.

#### Electrically isolated crystal: bipolar electron-hole screening

The screening of H-H and T-T sCDWs by electrons and holes, respectively, was addressed in refs. 18,23. In the case of electron-hole screening in typical perovskite ferroelectrics, the main source of compensating free carriers is electron transfer from the valence to the conduction band over the bandgap of the ferroelectric^23^. For a 180° wall (Fig. 1c,d) with 2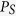 bound charge per unit area, where 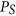 is the spontaneous polarisation, the screening requires 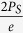 (*e* is the elementary electron charge) free carriers per unit area. Thus, the formation energy of a single wall in a sample can be evaluated as^23^:


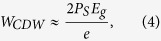


where 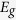 is the bandgap (one should note that in the case of a domain pattern of sCDWs Eq. (4) gives the energy of a pair T-T/H-H sCDWs). For a bandgap of about 3 eV, which is typical for perovskites, the energies of sCDWs are some two-order of magnitude larger than the energy 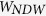 of neutral DWs, i.e.^23^


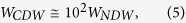


while the energy of NDWs is, on the lines of Landau theory^31^:


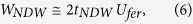


where 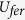 is the gain of the energy density of the ferroelectric phase with respect to the paraelectric one and 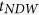 is the half-width of the NDW.

The carrier concentration in the wall can be evaluated as


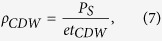


where 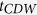 is the half-width of the sCDW. Taking 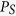 = 0.3 C/m^2^ and 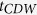 = 10 nm, one finds 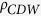 = 2⋅10^26^ m^−3^. Such a concentration vastly exceeds any realistic intrinsic equilibrium concentration of free carriers in nominally insulating ferroelectrics. Based on this fact, Gureev *et al.*^23^ concluded that the participation of equilibrium carriers in the screening can be neglected. Thus, an important feature of this screening scenario is that sCDW formation is virtually insensitive to the equilibrium free carrier concentration (and conductivity) in the single-domain material, be it a pure or a moderately doped material.

#### Electrically isolated crystal: Unipolar screening

One may conceive an alternative screening scenario; Surowiak *et al.*^7^, discussing formation of a sCDW in a PbTiO_3_ crystal, argued that the free charge needed for screening of a single sCDW in a crystal can be “collected” from its volume. According to this scenario, electron transfer across the bandgap is not needed. The realisation of this scenario is limited even when the amount of free carries in the crystal suffices for screening of the bound charge of the wall. The point is that, for not too small crystals, the needed amount of free carriers can be available only due to the presence of doping impurities in the material. Once the free carriers concentrate at the walls, the space charge of the ionised impurities in the bulk implies a very strong increase of the energy of the system. (See discussion in Supplementary Information Note 1)

#### Electrically isolated crystal: Screening with photon generated carriers

The energy of sCDW formation by electron transfer across the bandgap may be large, but this energy can be naturally provided by externally supplied superbandgap photons which directly generate electron-hole pairs. In this scenario, sCDWs form once the required number of free carriers has been provided. In practice electron-hole pairs can be provided by illumination with ultraviolet light.

#### Electrically isolated crystal: Mixed electron/ion screening

As pointed out above (see also Supplementary Information Note 1), screening of sCDW through the collection of free equilibrium carriers from adjacent domains leads to very high sCDW formation energy due to the space charge of the ionised impurities in the bulk of the crystal. However, this energy is substantially reduced if the ionised impurities are mobile, i.e. if they can be redistributed during the formation of the sCDW. In this case, the mobile carriers have both polarities, and the situation resembles the electron-hole screening discussed above. For example, in the case of an n-type material, the equilibrium electrons in the conduction band can screen the positive bound charge while the ionised donors can screen the negative bound charge. If the total amount of charge in the crystal is large enough to neutralise the bound charge at the walls, such a screening mechanism is energetically more favourable than the pure electron-hole screening since no energy penalty is paid for the electron transfer across the bandgap. In this case, the sCDW formation energy, 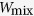, can be evaluated as the energy associated with the deviation of the polarisation in the wall region from its spontaneous value (c.f. the discussion from ref. 23), i.e.


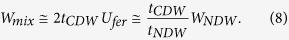


To get this equation, Eq. (6) was used. Note that Eq. (8) is not valid for ferroelectrics near the rotational phase transition in which the formation energy of anomalously thick CDWs^32^ is even smaller.

For typical perovskite ferroelectrics, 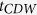 is expected to be one order of magnitude larger than 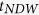
23. Thus, comparing the estimate of Eq. (8) with Eq. (5), we see that in perovskite ferroelectrics, sCDW formation with a mixed electron-ion screening mechanism is more favourable energetically than bipolar electron-hole screening.

An essential feature of this screening scenario is that, for given charge densities of mobile carriers of both signs, 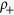 and 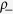, and the component of spontaneous polarisation normal to the walls, 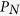, the average domain width should exceed a certain minimum value 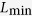. Relation between these parameters can be found from the condition that all mobile carriers of the material are used for the screening of sCDWs:


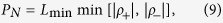


yielding


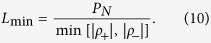


Two more characteristic features of the mixed screening mechanism are evident. First, it requires substantial redistribution of the ionised impurities, implying a relatively slow sCDW formation. Second, a strong difference in the conduction of H-H and T-T walls is expected. In view of their much higher electronic mobility, the walls screened by electronic carriers will exhibit substantially higher conductivity than those screened by the significantly less mobile charged defects.

Experimental data on sCDWs in bulk crystals of BaTiO_3_^5^, strongly suggest that it is the mixed screening mechanism which takes place in this system. Here, H-H 90° sCDWs artificially created in the materials were found to be highly conductive (10^9^ times more than the domains), while T-T sCDWs did not exhibit increased conductivity. This situation perfectly matches with the typical n-type conduction in a material in which oxygen vacancies (Vö) play the role of donors. Since the mobility of the latter is much smaller than that of electrons, the T-T walls screened with Vö do not exhibit measurable conductivity contrast with respect to the domains themselves.

#### Screening of sCDW with charge provided from external source

Charge injection from the crystal exterior may strongly reduce the formation energy of sCDW^18^,^23^,^33^. Depending on the band gap of the ferroelectric and on the ferroelectric/metal work function difference, the energy penalty associated with the carrier generation is necessarily reduced compared to the electron transfer over the band gap either for electrons or holes^18^,^23^,^33^. For a certain combination of these parameters, the energy penalty can be even negative, implying negative formation energy of sCDW^34^,^35^. Estimates show that in BaTiO_3_ with Pt electrodes, T-T sCDWs should have negative formation energy, thus forming spontaneously^23^,^34^,^35^. Such a phenomenon has never been documented experimentally, which can be explained by surface effects associated with surface states and surface termination^23^ not taken into account in the model. Mobile charged defects like oxygen vacancies Vö due to oxygen loss from the sample may give similar effects to those resulting from the electronic screening discussed above. The formation of H-H and T-T configurations supported by purely ionic compensation has been documented for LiNbO_3_ and LiTaO_3_ crystals (see^36^ and references therein).

### Domain walls in poled state in domain engineered BaTiO_3_

A possible way to obtain sCDWs is the so-called frustrative poling^37^,^38^, i.e. cooling the crystal through its ferroelectric phase transition with a dc field applied along a direction different from the permitted spontaneous polarisation directions. Such poling results in domains having several polarisation states whose projections onto the direction of the field are collinear with the latter. Below we describe the domain walls which can be created with frustrative poling in the fields of [110]_c_ and [111]_c_ orientations in classical tetragonal BaTiO_3_ single crystal; the suffix “c” indicates that Miller indices are given in the pseudo-cubic reference frame. Here we consider structures with only quasi parallel domain walls which are expected theoretically and also were mostly observed in our experiments.

#### [110]_c_ poling

**Figure 3:**
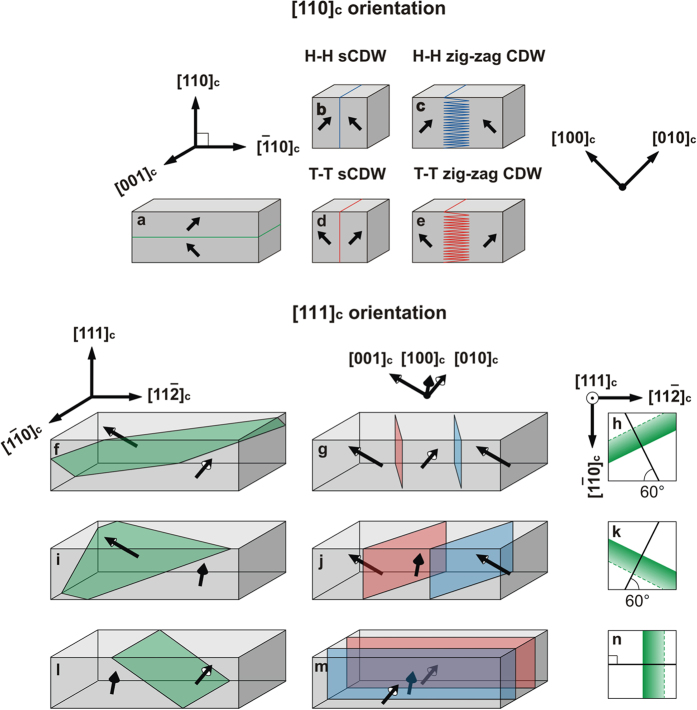
Domain states and 90° domain walls in BaTiO_3_ obtained through frustrative poling. (**a**–**e**) schematic views of domain states and domain walls in a [110]_c_ poled tetragonal BaTiO_3_ crystal observed from the [001] direction: neutral domain wall (**a**), planar H-H sCDW (**b**), zig-zag configuration of H-H sCDW (**c**), planar T-T sCDW (**d**), zig-zag configuration of T-T sCDW (**e**). (**f–n**) schematic views of domain states and domain walls in a [111]_c_ poled tetragonal BaTiO_3_ crystal: three possible orientations of NDWs (**f,i,l**); three possible orientations of sCDWs (**g,j,m**) with H-H and T-T configurations; (**h,k,n**) top views of all possible 90° DWs in the poled state for DW identification. Green stripes depict inclined NDWs while black solid lines represent sCDWs which are normal to the surface. Arrows show the directions of spontaneous polarisation.

Two domain states, with polarisation along [100]_c_ and [010]_c_ can coexist in a (110)_c_ plate poled along the [110]_c_ direction (Fig. 3a–e). These two domain states, differing in both ferroelectric and ferroelastic order parameters can be separated by (110)_c_ NDWs lying in the plane of the plate, Fig. 3a, and by 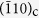 sCDWs that lie across the sample thickness^6^. The sCDWs have either H-H or T-T configurations (Fig. 3b,d), respectively. Sometimes zig-zag configurations (Fig. 3c,e) are also seen.

#### [111]_c_ poling

In the case of a (111)_c_ plate of a tetragonal BaTiO_3_ crystal poled along the [111]_c_ direction, three ferroelectric/ferroelastic domain states are equally preferred, separated from one another either by sCDW or NDW (Fig. 3f–n)^6^. In total, six domain walls (three sCDWs and three NDWs) are possible in a fully poled [111]_c_ crystal. The allowed orientations of sCDWs are 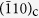, 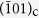, 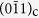 (Fig. 3g,j,m) and of NDWs are (110)_c_, (101)_c_, (011)_c_ (Fig. 3f,i,l). SCDWs are always perpendicular to the (111)_c_ surface while NDWs are always inclined. All walls have different orientations of their crossing lines with the large (111)_c_ face of the plate as seen from Fig. 3f–n. This readily enables the identification of the walls. Zig-zag shaped domain walls can also appear in (111)_c_ BaTiO_3_ crystal as a tilted version of zig-zag walls in (110)_c_ crystal, discussed above.

### Propitious conditions for formation of sCDW patterns

Here we address the conditions that allow controlled formation of sCDWs.

#### Material requirements

Both screening mechanisms, the bipolar electron-hole screening and the mixed electron-ion screening, support the formation of periodic patterns of sCDW. However, the formation energy of sCDWs screened by electrons and ions is smaller than that of sCDWs screened purely by electrons and holes (see Eqs. (5) and (8)), i.e. the electron-ion scenario is favoured if material composition allows. The main requirement for the electron-ion screening is obviously the presence of mobile ionised donors in the material. Alternatively it could be hole-ion screening and the presence of mobile acceptors.

In perovskite ferroelectrics like BaTiO_3_, positively charged oxygen vacancies Vö are relatively mobile and shallow donors. The ionised Vö can therefore accumulate on tail-to-tail sCDW where they screen negative polarisation charge, while electrons easily liberated from Vö can screen the positive polarisation charge at head-to-head sCDW. Since the total amount of Vö is always limited, the minimal period of sCDWs will be inversely proportional to the initial mobile free charge concentration (see Eq. (10)). Therefore, a high concentration of mobile impurities such as Vö will favour the formation of dense sCDW patterns. The mobile dopants can be introduced during crystal growth or by post-growth annealing in an oxygen deficient atmosphere. We keep in mind that a high concentration of mobile and shallow level dopants implies enhanced bulk conductivity, reducing the contrast between the domain and sCDW conductance.

In principle, the problem of the increased bulk conductivity inherent for electron-ion scenario can be circumvented if the sCDW formation is controlled by electron-hole screening. In this case, the doping level has a smaller or no impact on sCDW formation^23^ and the minimal domain size is controlled by other factors. Specifically, as it was shown by Ivanchik^33^, the minimal domain width 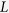 in electron-hole screening scenario is controlled by the condition that the residual depolarisation field


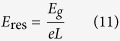


is smaller than the thermodynamic coercive field. This condition, in case of BaTiO_3_ with a forbidden gap of 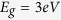, sets the minimal domain size limit at 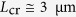
20. Thus, in terms of the electron/hole scenario such a domain size can be achieved independently from the Vö concentration. However, this scenario penalises the formation of sCDW by the relatively high energy cost of electron transfer across the band-gap. This may severely complicate sCDW formation on a practical level. The energy penalty, however, can be reduced by artificial electon-hole generation induced by superbandgap illumination, for example.

Note that we excluded scenarios which involve injection of mobile charge carriers into the sample from its exterior. The reason is that the external charge injection usually provides only one type of charge carriers which leads to formation of only one type of sCDW^23^,^34^,^35^,^36^. In contrast, screening by internal mobile charges fundamentally supports the formation of periodic sCDW structures, because an equal amount of charges of both polarities has to be employed to keep the sample’s neutrality.

Finally, note that a similar limit for the minimal sCDW period as in the case of electron-hole screening (which is related to the depolarisation field across a domain) also restricts the sCDW period in the electron-ion scenario. However, in the latter scenario, the residual depolarisation field is somewhat smaller than that resulting from electron-ion screening. For example, results by Sluka *et al.*^5^ for room temperature BaTiO_3_ imply 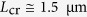. This value may be further reduced via an enhancement of the thermodynamic coercive field of the material. Such an enhancement leading to a reduction of 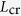 is feasible for BaTiO_3_ by using Sr-for-Ba substitution or by using other ferroelectrics (see Supplementary Information Note 2).

All in all, we distinguish two different approaches for the engineering of high density patterns of sCDW: (i) The use of a non-stoichiometric material where a high concentration of mobile ionised impurities is available (ii). The use of nominally stoichiometric materials where the frustrative poling is accompanied with artificial electron-hole generation by superbandgap illumination. The two approaches can be combined.

#### Principles of poling conditions

In principle, cooling through the phase transition in the absence of an electric field may result in the appearance of sCDWs. However, a controllable way of creation of patterns of sCDWs consists of cooling through the phase transition in a frustrative electric field, (“frustrative poling”)^5^,^37^. The primary role of the frustrative field is a reduction of the number of preferable polarisation states in the sample, which increases the probability of the appearance of 90-degree sCDWs.

The applied dc field also plays another role in sCDW formation; it reduces the polarisation component perpendicular to the sCDW (see Supplementary Information Note 3). Thus, the amount of bound charge at the wall is smaller and consequently the amount of mobile carriers needed for screening is smaller too. Since the screening in a nominally insulating (poorly conductive) material is a slow process, especially in the case of the electron/ion scenario, the smaller amount of screening charge needed can be more easily delivered to the wall. Thus, a dc-field-controlled reduction of the bound charge density at the wall favours formation of sCDWs. For [110]_c_ and [111]_c_ orientations of the poling field, our calculations show (see temperature-field diagram of BaTiO_3_ in Supplementary Information Note 3) that the aforementioned polarisation component normal to the sCDW is a decreasing function of the poling field, disappearing at field values of *E*_[110]_ = 2.8 kV/mm and *E*_[111]_ = 11.4 kV/mm, respectively. These field values correspond to the tricritical points for the transition having the normal-to-the-field component of the polarisation as the order parameter. The ideal situation for sCDW formation is a slow cooling in a field that is larger than the aforementioned critical values, thus passing through the second order phase transition where the normal component of the polarisation and therefore the bound charge at the wall appear continuously. According to our experience, the application of such a high electric field is hardly possible because of cracking and/or breakdown problems. At the same time, the application of maximal possible poling field favours sCDW formation, because of the reduction of the bound charge appearing at the wall at the transition.

### Formation of strongly charged domain walls in BaTiO_3_

Five types of BaTiO_3_ samples were studied, exhibiting a 5 order-of-magnitude spread of bulk conductivity (see Methods). The variation in conductivity, accompanied by colour variations, originated mostly from differences in Vö concentration. sCDWs were obtained under frustrative poling procedures in both (110)_c_ and (111)_c_ plates. To reduce probability of cracking, the poling field was kept sufficient to reduce the polarisation jump during the phase transition but not too elevated. Details on the samples, their preparation procedures and their poling processes are given in Methods.

#### Identification of strongly charged domain walls

Two optical methods were used for the identification of sCDWs, the observation of domain structures in polarised light between crossed polariser and analyser (named hereafter “polarisation analysis”) and the so-called “intensity analysis”, based on the examination of intensity variation of the transmitted light along the domain walls. Application of these methods becomes possible when the out-of-plane component is the same for all domains and specified. This can be achieved when the poling electric field strongly exceeds the coercive field. Samples were exposed with an electric field up to 20 kV/cm that is 20 times higher than coercive field (1 kV/cm). Thus, the orientation of the out-of-plane component of polarisation in all domains is strictly specified. Identification of CDWs was performed under applied electric field as well as in zero field. This fact allows us to conclude that the out-of-plane component of polarisation is the same in all domains even in the absence of an electric field. All obtained CDWs are 90-degree domain walls; this is clear from polarisation analysis (see Supplementary Information Note 4). For the verification of the out-of-plane polarisation component see Supplementary Information Note 6.

**Figure 4:**
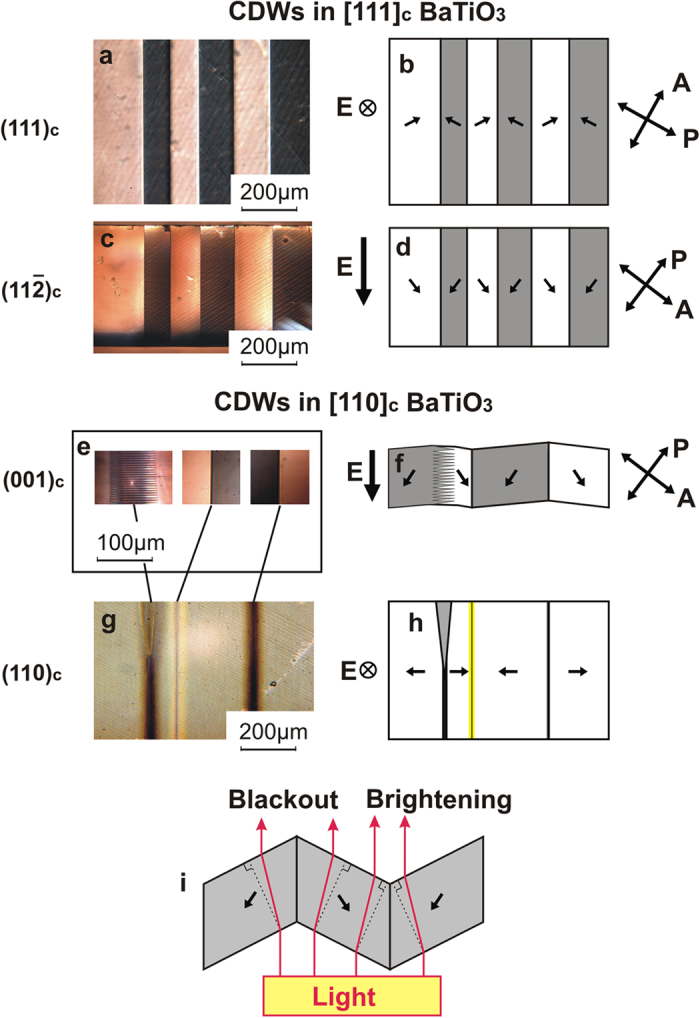
Identification of sCDW using the polarisation and intensity analysis methods. Micrographs taken with polarising microscope (**a,c,e**) and the corresponding schematics (**b,d,f**). Arrows labeled “A” and “P” show the orientations of the analyser and polariser, respectively. (**a–d**) and (**e–h**) correspond to (111)_c_ and the (110)_c_ cuts of the plate, respectively. The orientations of the poling field (symbols marked with E), the crystallographic planes of the micrographs, and the direction of the spontaneous polarisation (non-labelled arrows) are indicated. The intensity analysis: the light intensity map (**g**) and its schematics (**h**). The nature of the contrast in the intensity analysis is explained in (**i**). In (**e–h**) a zig-zag wall is seen.

The application of polarisation analysis to a (111)_c_ oriented BaTiO_3_ plate is illustrated for the (111)_c_ top view in Fig. 4a (image) and 4b (scheme) and for the 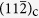 side view in Fig. 4c (image) and 4d (scheme). Details of polarisation analysis are presented in Supplementary Information Note 4. For a plate of this orientation, once the direction of the poling field is known, the information about the orientation of borders between the images of the domains and the optical indicatrices inside the domains enable the full identification of the domain walls.

For (110)_c_ oriented BaTiO_3_ plates, the identification of the domain walls can be done using the (001)_c_ side view (Fig. 4e,f). On the contrary, using solely the top view is insufficient as there is no misalignment of the optical indicatrices of the domains (c.f. the orientation of the polarisation in Fig. 4h, which controls the indicatrix orientation). Note that for the (001)_c_ side view (Fig. 4f), the disorientation of the optical indicatrices is controlled by a small “clapping” angle (the angle between the identical pseudo-cubic directions in the neighbouring domains), which is, however, sufficient for an accurate experiment.

“Intensity analysis” is based on the refraction of light in a crystal deformed by the spontaneous ferroelastic strains. As shown in Fig. 4i, in a multi-domain plate, the surfaces of neighboring domains are not parallel. As a result, the light propagating alongside the walls either concentrates or diverges at the walls (Fig. 4i) leading to the corresponding modulation of the observed light intensity.

The application of this method to a (110)_c_ oriented plate is illustrated in Fig. 4g,h. For such a plate orientation, once the orientation of the poling field is known, the intensity analysis provides the full identification of sCDWs. In this situation, this method is very useful for *in-situ* sCDW identification during poling, because the sample cannot be easily observed from two directions at the same time. For (111)_c_ oriented plates, the intensity analysis can also be applied for the sCDW identification. However, observation from both (111)_c_ and 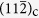 faces is necessary, which is not suitable for *in-situ* sCDWs identification during poling.

A combination of the two identification methods is suitable for the *in-situ* sCDW identification in (111)_c_ oriented plates (Fig. 4a). Here the orientations of the walls are found by polarisation analysis while differentiation between H-H and T-T walls is obtained using the intensity analysis through presence or absence of bright lines as seen in the same figure. Both methods also enable visualisation of zig-zag structures seen in Fig. 4e,f (see Supplementary Information Note 5).

#### Influence of the material’s non-stoichiometry on charged domain wall spacing

Samples of five types labeled as S1-S5, prepared with different oxygen non-stoichiometry (see Methods) were used for this study. They differ in colors (colorless, dark-yellow, light-brownish, greenish and grey) corresponding to a 5 order-of-magnitude spread of free charge density. All samples were (111)_c_ oriented 5 × 1.5 mm^2^ plates of 250 μm thickness with the long edge parallel to the 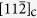
direction. They were poled, following the procedure described in Methods. The experiments were conducted using samples exhibiting only 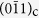
orientation of sCDWs.

**Figure 5:**
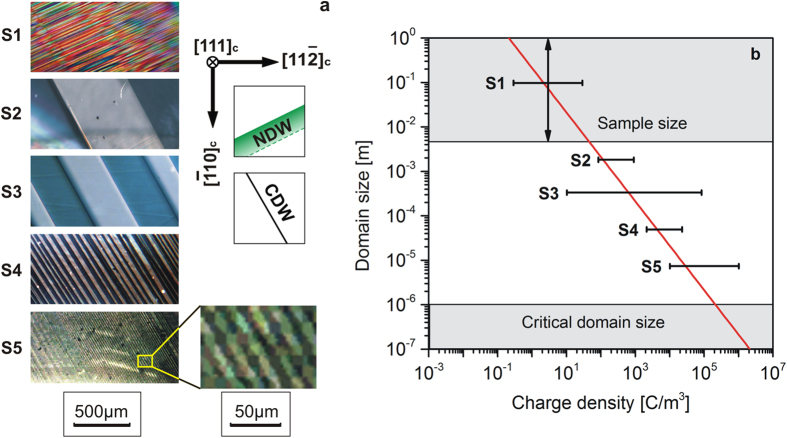
Charged domain wall period in (111)_c_ BaTiO_3_ crystal plates with different charge density. (**a**) Optical micrographs of domain patterns with drawings of allowed orientations of domain walls: CDW–strongly charged domain walls, NDW – neutral domain walls; (**b**) Dependences of the domain size versus charge density in a single-domain material–estimated charge density from the literature for (S1, S3, S5)^40^,^41^ and from our experiments (S2, S4, S5); red line – the minimal domain size as a function of the mobile charge density in the single-domain material as predicted by the electron/ion screening scenario according to Eq. (12); upper horizontal line – the sample size; lower horizontal line – minimal domain size consistent with the stability of ferroelectricity in the domains, evaluated using the results from ref. 5. The calculation of the error bars is described in Supplementary Information Note 8.

In samples with the lowest non-stoichiometry, S1, NDWs or zig-zag configurations were observed, while no sCDWs were identified; it is seen from Fig. 5a. In contrast, in samples with the highest non-stoichiometry, S5, the domain patterns consisted exclusively of sCDWs having spacing of several microns (Fig. 5a.) Samples with intermediate non-stoichiometry (S2-S4), exhibited planar sCDWs with spacing decreasing as the free charge content increased. Typical domain patterns are shown in Fig. 5a. The averaged domain size vs. the free-charge density in the single-domain material is plotted in Fig. 5b.

The results fit the scenario of mixed electron/ion screening discussed above and in ref. 5. Coming from the theoretical approach that considers the mixed electron/ion screening scenario we assume an ideal case with planar parallel sCDWs, excluding special cases e.g. zig-zag domain walls, domain walls deviating from their crystallographic orientations due to pinning or inhomogeneous force fields, etc. As reported earlier^5^, CDWs in BaTiO_3_ crystals demonstrate strongly elevated conductivity. We confirmed this effect by measuring current through the crystal with and without CDWs (see Supplementary Information Note 7 and Fig. S8). We observe that the conductivity of initially single-domain crystal increases proportionally to the number of created CDWs. When considering that the cross-section of a CDW (~10 nm × 1.4 mm) is approximately six orders of magnitude smaller than the sample cross-section (~5 mm × 1.2 mm), the conductivity of an individual CDW must be also over six orders of magnitude larger than the bulk conductivity. Therefore, the combination of the known polarisation states, orientation of CDWs, and the observed conductivity, is the evidence of sCDWs as classified in part “*Classification of Charged Domain Walls*”.

To substantiate our analysis, we evaluated the minimal domain size of the sCDW pattern, 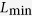, in terms of this scenario, using Eq. (10). For our 90° pattern, we set the component of the spontaneous polarisation normal to the sCDW 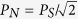 and assume all free electrons are produced by mobile donors, so that 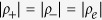 where 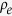 is the charge density of free electrons in the single-domain material. Then Eq. (10) yields


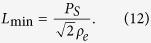


Equation (12) is plotted with the red line in Fig. 5b, using, for BaTiO_3_ at room temperature, 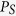 = 0.26 C/m^2^. We also show in this figure the upper limit for the domain spacing in a finite sample – the maximal crystal dimension. The second horizontal line in this figure gives the lower limit for domain spacing in a BaTiO_3_ crystal associated with the destabilisation of ferroelectricity by the residual depolarisation field^33^ as discussed above, in the Section “Materials requirements”.

The patterns shown in Fig. 5a can be interpreted, based on Fig. 5b, in terms of the mixed electron/ion screening scenario. In Fig. 5b, for each type of sample, the average domain size and the charge density 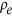 are indicated. The arrowed line in Fig. 5b means that we do not observe sCDWs in S1 samples, but in principle, in such a material a domain size larger than the size of our samples cannot be excluded. The absence of sCDWs in S1 sample is consistent with its low free charge content, i.e. low 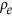. Specifically, as seen in Fig. 5b, in S1 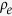 is around 1 C/m^3^. According to the red line, this density corresponds to the minimal domain size of some 10 cm that significantly exceeds the size of the sample.

As for samples S2–S5, they belong to the “allowed” interval of the domain size. The domain sizes in these samples are consistent with the mixed electron/ion screening scenario. Indeed, for these samples, the right-hand ends of the charge-density error bars are above the red line, implying that free-charge density in single-domain materials suffice for the screening of sCDWs with the observed periodicity.

#### Influence of poling field on spacing of sCDWs

**Figure 6:**
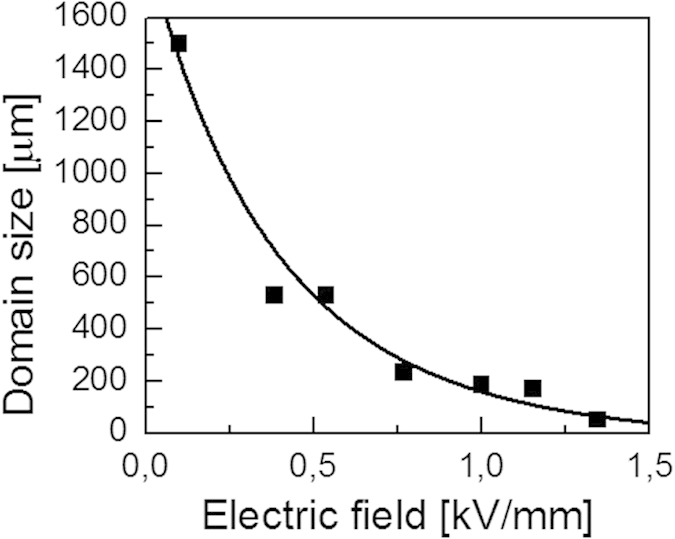
Domain size versus electric field applied during frustrative poling procedure. Data extracted from charged domain patterns in (111)_c_ S4 BaTiO_3_ crystal plates under the poling field at 80 °C.

As discussed above, the role of the frustrative poling field is two-fold: reduction of the number of favourable domain states and reduction of the bound charge appearing at the wall at the transition. Thus, the stronger the poling field, the smaller the bound charge and the easier it can be screened, favouring the sCDW formation. The implication is that a high poling field may support the formation of sCDW patterns of higher density. This trend agrees qualitatively with our experimental observation (Fig. 6). However, the effect seems to be stronger than one can expect from simple arguments.

## Conclusion

In this work we addressed theoretically and experimentally the controlled formation of domain patterns with charged domain walls in a classical proper ferroelectric. Based on the results we identified the mixed electron/ion screening scenario as a favourable governing mechanism for the formation of charged domain wall patterns. Using direct optical observations we established the dependencies of the average domain size on the non-stoichiometry (free charge concentration) and on the magnitude of the frustrative poling field. The data obtained are in agreement with the limitations imposed by the mixed electron/ion screening scenario. We observed domain widths in the range 7–1500 μm, depending on the degree of non-stoichiometry and strength of the poling field. The minimal domain size was not conditioned by any intrinsic limitation of the material. Neither are there any restrictions which would interfere with its reduction down to the minimum set by the polarisation stability limit in the domains^33^, which for BaTiO_3_ at room temperature is evaluated as about 1 μm^5^,^20^. Even this limit is not absolute; it can be pushed down by increasing the thermodynamic coercive field of the material. This is possible by compositional modification of the material, e.g. via a Sr substitution for Ba. The formulated principle and concepts are general and applicable to any proper ferroelectric having non −180° domain walls, thus the road is opened for the exploitation of the unique properties of charged domain walls in ferroelectrics.

## Methods

BaTiO_3_ single crystals studied in the present work were obtained from the commercial supplier “GB group Inc.” ( www.crystalsland.com) as a nominally pure material grown by top seeded solution growth method (TSSG). From these crystals, five types of BaTiO_3_ samples, differing in colour and conductivity (Table 1) were prepared. The first one, S1, is a pure crystal annealed for 10 hours in oxygen at 1200 °C – transparent colourless crystal with a low conductivity. S2 is a transparent dark-yellow crystal from a separate batch. S3 and S5 were prepared from the same batch as S1 by means of annealing in vacuum 2⋅10^−5^ mbar at 1200 °C during 36 hours (transparent light-brownish crystal) and 170 hours in 1⋅10^−5^ mbar vacuum at 1200 °C (transparent grey crystal) respectively. Thus, S3 and S5 crystals were intentionally oxygen depleted with different level of oxygen depletion. S4 is a transparent greenish crystal also from the separate batch. This crystal changed colour to brown or grey after poling. In ref. 39 such electro-coloration is considered as one of the attributes of migration of oxygen vacancies existing in the crystal. The difference in colour and conductivity between the described samples is related to the difference in the concentration of oxygen vacancies.

Crystals were shaped in (110)_c_ oriented 5 × 5 × 0.25 mm plates (S1, S2 crystals) and (111)_c_ oriented plates 5 × 1.2 × 0.25 mm with the edge of the largest dimension along the 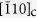 and 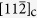 directions (S1, S2, S3, S4, S5 crystals). The accuracy of deviation of main surfaces from the parallel state is ± 0.06°. Transparent platinum electrodes 10 nm thick were deposited on the main surfaces of the samples. It allowed observation of the domain structure during poling and gave possibility of *in-situ* identification of domain structure.

Conductivity of the pre-treated crystals (S1, S3, S5) was estimated according to literature data^40^,^41^. Graphs of conductivity vs. partial pressure of oxygen presented in this work were used to obtain the conductivity of our samples (see Supplementary Information Note 8). From these graphs intervals of conductivity corresponding to partial pressure of oxygen in the furnace during the annealing of each sample were estimated.

For samples S2, S4 and S5 conductivity was obtained experimentally. It was measured by applying voltage and measuring current at 300 °C after more than 30 hours of current stabilisation. For conductivity measurements, 100 nm thick platinum electrodes were deposited on the main surfaces of the samples. Under the assumption that the conductivity is governed by free electrons, the concentration of the latter was evaluated using electron mobility in BaTiO_3_ as calculated from the relation^42^:


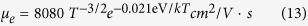


Based on the electron concentration in the samples indicated by conductivity σ, we have estimated the free charge density (see Table 1):


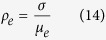


### Frustrative poling

#### [110]_c_ field poling

**Figure 7:**
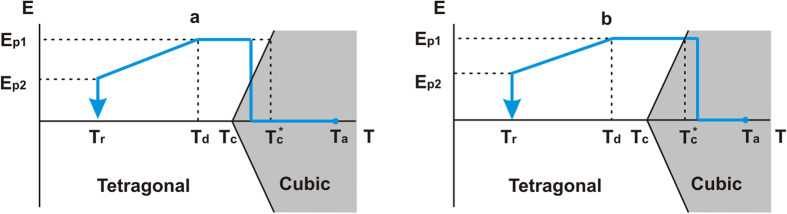
Poling procedures in an electric field parallel to the [110]_c_ (a) and [111]_c_ (b) directions. The path is indicated by blue arrows. 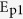, 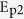 – poling electric fields, 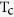
– zero field PT temperature, 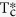 – E-field induced PT temperature, 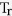
– room temperature, 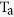
– temperature of annealing, 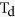
– temperature at which the slow decrease of the electric field began.

The most reliable sCDWs formation was achieved by a field-induced phase-transition (E-field PT) (Fig. 7a). Firstly, temperatures 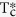 where E-field PT is induced (for 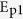 = 1.2 kV/mm) and 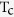 where PT occurs at zero E-field were identified as 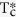 = 130.2 °C and 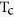 = 129.5 °C, respectively. Next, the samples were heated to 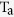 ≈ 160 °C, 30–40 K above the ferroelectric-paraelectric PT. At this temperature, the samples were annealed for ~30 minutes and subsequently cooled. The cooling rate was kept at 0.1 °C/min when approaching the PT temperature. At a temperature ~0.1 °C below 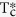, 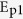 = 1.2 kV/mm was applied, which induced the PT and formation of a periodic structure with the 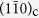 planar sCDWs. Then the sample was slowly cooled with temperature rate of 0.1 C/min to 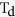 = 120 °C. To prevent cracking and high current because of high sCDW conductivity, subsequent cooling to room temperature 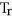 was accompanied with slow decrease of electric field to 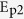 = 0.5 kV/mm. Sometimes the 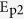 value was lower because of high current through sCDWs. The cooling rate was kept around 1 °C/min. Finally, the samples were kept several hours under an electric field. All processes were observed with a polarising microscope.

#### [111]_c_ field poling

The [111]_c_ field poling was done by crossing the transition line on cooling (Fig. 7b). For samples (S1–S4), first, the temperatures of PT on cooling at zero electric field (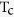 = 128.6 °C) and of the E-field-induced (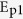 = 1.2 k V/mm) PT (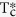 = 129.5 °C) were identified. Next, the samples were heated up to 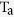 = 160 °C and annealed at this temperature for ~30 minutes. Then, the samples were cooled down with cooling rate of 1 °C/min to a temperature a few degrees above 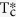 where an electric field of 1.2 kV/mm was applied. Under such an electric field the samples were cooled down through the phase transition to 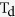 = 120 °C with a rate of 0.1 °C/min. The domain structure appeared in the temperature range from 129.5 °C to 127.2 °C as observed with the polarising microscope. Cooling from 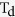 to 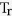 was accompanied with a slow decrease of the electric field to 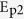 = 0.5 kV/mm. The cooling rate was around 1 °C/min. Samples were then kept several hours at room temperature under the exposure of an electric field of 0.5 kV/mm.

For the strongly oxygen depleted crystals S5, a poling procedure different from that outlined above was used. Because of high current, the poling electric field E_p1_ was reduced down to 0.1 kV/mm and the poling stopped at around 100 °C since the domain structure becomes unstable at lower temperatures.

The rate of successful preparation of CDWs was roughly 60–80% for S2, S3 and S4 crystals. For the crystal S5 we do not have a reliable statistics. Due to its higher conductivity, poling attempts were often accompanied by breakdown, particularly at the phase transition. The main reason of failures in CDWs preparation was related to cracks and breakdowns during poling.

### Shape of the samples

**Figure 8:**
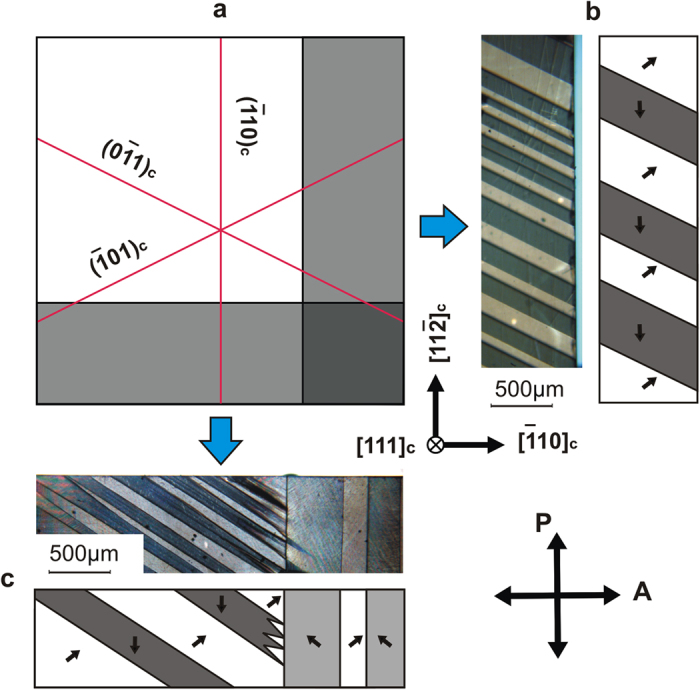
SCDWs in [111]_c_ oriented BaTiO_3_ single crystal. Schematic of an [111]_c_ oriented BaTiO_3_ plate (**a**) and two rectagrular samples (**b,c**) cut from it. The orientations of possible sCDWs are shown in (**a**) with solid red lines. Micrographs taken with a polarisation microscope (orientations of the analyser and polariser are shown with arrows) with the coresponding schematics (**b,c**). The micrographs are taken when a poling electric field of 0.5 kV/mm is applied.

To observe formation of sCDWs of every orientation, two different cuts of (111)_c_ crystal plates were studied, with the long edge parallel to the 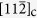 (Fig. 8b) and to the 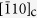 (Fig. 8c) directions, which will be hereafter referred to as 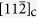 and 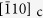-samples, respectively. In general, there are three possible orientations of sCDWs in such samples (shown with straight lines in Fig. 8a). All obtained patterns were extended to the whole size of the sample and didn’t depend on sample size up to the maximum considered sample size of 5 × 1.2 mm.

In 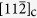-samples, sCDWs of one of the two, 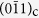 or 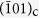, orientations were usually observed. In Fig. 8b, the example of 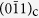 oriented sCDWs is presented. In contrast, in 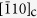-samples, typically two types of walls coexist: one of the 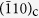 orientation and the other either of the 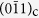 or of the 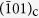
orientation. Figure 8c shows the situation where the walls of the 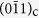 and 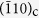 orientations coexist in a 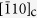-sample. Sometimes intersections between sCDWs were detected (Fig. 8c); however, at all intersections the domains are distorted.

The images of domain patterns shown in Fig. 8 where taken under a poling electric field 0.5 kV/mm. Once the poling field is off, the patterns change leading to the partial annihilation of sCDWs of opposite polarities. We found that the most stable domain walls are the 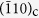
walls in 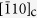-samples, the walls in 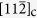-samples are less stable while 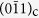 and 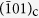 sCDWs in 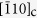-samples are the least stable. We also found that the smaller the domain width, the less stable the domain pattern.

## Additional Information

**How to cite this article**: Bednyakov, P. S. *et al.* Formation of charged ferroelectric domain walls with controlled periodicity. *Sci. Rep.* **5**, 15819; doi: 10.1038/srep15819 (2015).

## Supplementary Material

Supplementary Information

## Figures and Tables

**Table 1:** Characterisation of the samples.

Sample	Colour	Conductvity [S/m]	Charge density [C/m^3^]
S1	Colourless	10^−5^–10^−3^	10^−1^–10^1^
S2	Dark-yellow	10^−2^–10^−1^	10^2^–10^3^
S3	Light-brownish	10^−3^–10^1^	10^1^–10^5^
S4	Greenish	10^−1^–10^0^	10^3^–10^4^
S5	Grey	10^0^–10^2^	10^4^–10^6^

Studied types of BaTiO_3_ crystals with their colour, conductivity measured at 300 °C, and the free charge density evaluated from the conductivity.
