# Supplementary material for: Formation of charged ferroelectric domain walls with controlled periodicity
Source: Sci Rep. 2015 Oct 30;5:15819. doi: 10.1038/srep15819 (PMC4626787; doi:10.1038/srep15819)
Supplement: Supplementary Information [file srep15819-s1.pdf]

Supplementary information

Formation of charged ferroelectric domain walls with controlled periodicity

Petr S Bednyakov, Tomas Sluka, Alexander K Tagantsev, Dragan Damjanovic, Nava Setter

SUPPLEMENTARY NOTES

Supplementary Note 1: Unipolar screening in isolated crystal

One might conceive a screening scenario where the free charge needed for the screening of a sCDW can be "collected" from the volume of the adjacent domains [7]. According to this scenario, electron transfer across the forbidden gap is not needed. In this scenario, the collection of the needed amount of charge is limited by the domain size and the free carrier concentration in the material. For example, for a 180° sCDW in a sample having the shape of a thin plate of thickness *2L* with the spontaneous polarisation normal to its plane depicted in Fig. S1, it is possible if the evident condition

$$P_S < L\rho_e \qquad \text{(S1)}$$

is met. Here ρ_e is the bulk free-carrier concentration in a single-domain sample. For a reasonably high ρ_e, e.g. for $PbTiO_3$ $\rho_e \cong 10^{24}\ \mathrm{m^{-3}}$ [7], this restriction is quite mild, requiring the crystal size to be just two orders of magnitude larger than the wall thickness. However, this scenario leads to enormous formation energy of sCDW due to space charge formation in the domains adjacent to the sCDW.

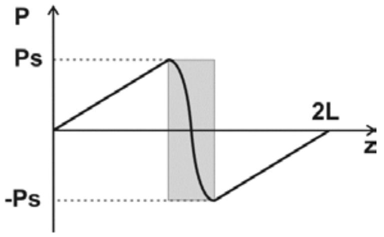

Supplementary Figure S1. Polarisation profile. The polarisation profile in a sample containing a 180° sCDW which is screened by the free electrons collected from the sample, leaving the space charge of ionised impurities. The sample has the shape of a thin plate of thickness *2L* with the spontaneous polarisation normal to its plane.

To show this, consider a crystal with a H-H sCDW in its middle (Supplementary Fig. S1). Once the free carriers are removed from the bulk of the domains to screen the bound charge at the sCDW, the domains acquire a space charge of ionised impurities equal to $\rho_{\mathrm{imp}} = P_S / L$, which must be smaller than the maximal density of the depletion charge ρ_e. This results in a longitudinal polarisation gradient [7][1]

---

[1] Here, following [23], we have neglected the difference between P and D to within $\frac{\varepsilon_f}{\varepsilon_b} << 1$ where ε_f is the dielectric permittivity of the ferroelectric.

$$\frac{dP}{dz} \approx divD = \rho_{\mathrm{imp}} = P_S / L\,. \qquad \text{(S2)}$$

In turn this gradient results in a strong deviation of the polarisation inside the domains from its spontaneous value. Specifically, such a gradient implies the vanishing of the polarisation at the side faces of the crystal (as shown in Supplementary Fig. S1). The energy penalty per unit area of the wall for this deviation can be evaluated (c.f. the estimates for the wall energy in the main text) as

$$W_{dep} \approx 2LU_{fer}\;. \qquad \text{(S3)}$$

Using Eqs (5), (6), and (S3), the formation energy of sCDW according to the “collection” scenario and that according to the electron/hole scenario can be compared, giving:

$$\frac{W_{dep}}{W_{CDW}} \cong \frac{L}{10^2 t_{NDW}}\;. \qquad \text{(S4)}$$

Keeping in mind that the half-width of neutral domain walls, t_{NDW}, in perovskites rarely exceeds 1-2 nm, Eq. (S4) shows that, except for nanometre-size crystals, the scenario associated with the collection of the free charge is energetically unfavourable.

Thus, the collection of free carriers for sCDW screening from the whole crystal is unrealistic as it corresponds to an even higher formation energy of sCDW than that of electron transfer over the forbidden gap (c.f. Eq.(5) from the main text).

Ionic species (e.g. oxygen vacancies) may also be considered for bound charge screening at sCDW. However, if only mobile charges of one polarity are available, the above discussion leading to Eq. (S4) is applicable, implying an enormous formation energy of sCDW. Note that the above considerations are valid for a situation where charge exchange between the crystal and the rest of the system is impossible.

Supplementary Note 2: Tailoring of thermodynamic coercive field.

Here we discuss the possibility to tailor the thermodynamic coercive field in perovskite barium titanate type ferroelectrics. An important feature of such ferroelectrics is that, under an electric field opposing the spontaneous polarisation, the latter may lose its stability either by changing its absolute value or by rotation [43]. In $BaTiO_3$ at room temperature the instability with respect to the rotation is decisive, controlling the thermodynamic coercive field. Qualitatively, this is related to an ease of polarisation rotation in the system, which can also be identified by a high dielectric anisotropic factor

$$\gamma = \frac{\varepsilon_a}{\varepsilon_c} \qquad \text{(S5)}$$

where ε_c and ε_a are the dielectric permittivity along and perpendicular to the spontaneous polarisation, respectively. Quantitatively, the thermodynamic coercive field is inversely proportional to γ [43]. This opens a possibility of controlling the coercive field by modification of γ. In $BaTiO_3$ at room temperature $\gamma = 30$ [20]. Such a high value is conditioned by the closeness to the tetragonal-orthorhombic phase transition. For the enhancement of the room temperature coercive field in $BaTiO_3$, one can use the Sr-for-Ba substitution. Such a substitution will lower the temperature of the tetragonal-orthorhombic transition [44], leading to a reduction of γ and the sought increase of the coercive field. A high thermodynamic coercive

field is also expected in other perovskites, which do not have the second ferroelectric phase, like $PbTiO_3$, where the value of γ is comparable to one.

Supplementary Note 3: Phase transition in $BaTiO_3$ in the presence of frustrated electric field

The problem of the phase transition of $BaTiO_3$ under a frustrated electric field was addressed by Bell [45]. He derived temperature-field phase diagrams for an electric field oriented along the $[110]_c$ and $[111]_c$ directions, as shown in Supplementary Fig. S2. The symmetries indicated in the diagrams correspond to those of the material in the absence of the electric field. Under the field the symmetry of the material in some of the regions in the diagram is evidently lower than that indicated, however, we will address the regions on the diagram using the latter. When preparing sCDW via cooling from the paraelectric phase under a frustrative poling field, we are interested in the cubic/tetragonal lines in the diagrams. At these lines, the order parameter of the phase transition is the component of the polarisation, which is normal to the field. This component can appear either smoothly or abruptly, corresponding to second and first order transitions, respectively, separated with a tricritical point. Using the same thermodynamic parameters as in Ref. [45], we evaluated the poling fields to be 2.8 kV/mm and 11.4 kV/mm, for fields parallel to the $[110]_c$ and $[111]_c$ directions, respectively (red points in Supplementary Fig. S2).

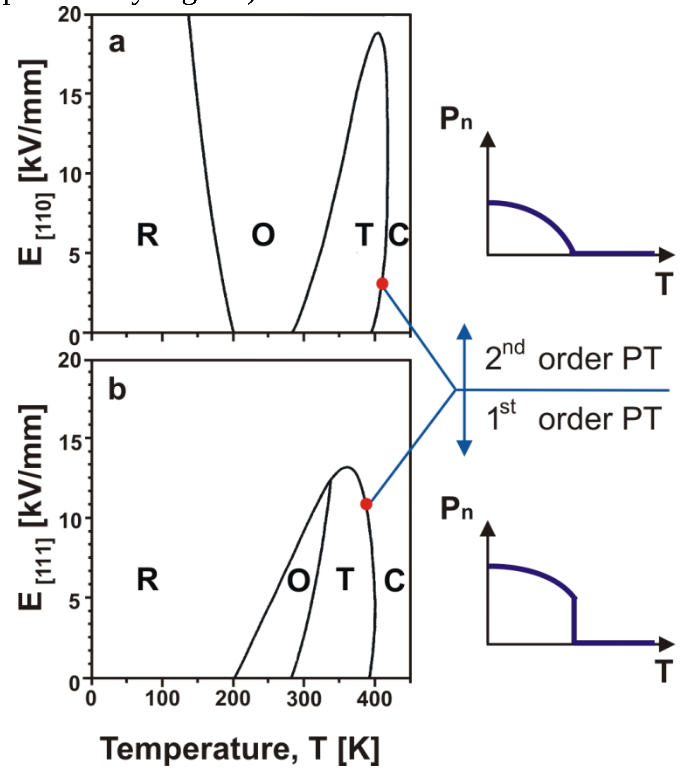

Supplementary Figure S2. Temperature – electric field phase diagrams. Phase diagrams of $BaTiO_3$ for poling fields parallel to the $[110]_c$ and $[111]_c$ directions. Transition lines are traced according to results of Ref. [45]. The positions of tricritical points, calculated using the thermodynamic parameters of $BaTiO_3$ from Ref. [45], are shown with red dots. Plots in the P_n, T coordinates show schematically the developments of the polarisation component normal to the direction of the field, in the cases of first and second order transitions.

Supplementary Note 4: Polarisation analysis

In Fig. S3, a method of identification of the in-plane orientation of the optical indicatrix is presented. 360-degree rotation of a typical $(111)_c$ oriented $BaTiO_3$ crystal with charged domain walls between crossed Nicols was performed. The crystal is split into domains of two different orientations of polarisation. Angles of rotation listed in Fig. S3 correspond to the full darkening of one type of domains (0°, 90°, 180°, 270° and 60°, 150°, 240°, 330°) and equal intensity of transmitted polarised light through both of the domains (30°, 75°, 120°, 165°, 210°, 255°, 300°, 345°). The optical indicatrix orientation corresponds to the polarisation vector orientation. This allowed us to identify directions parallel and perpendicular to the polarisation vector projections to the $(111)_c$ plane.

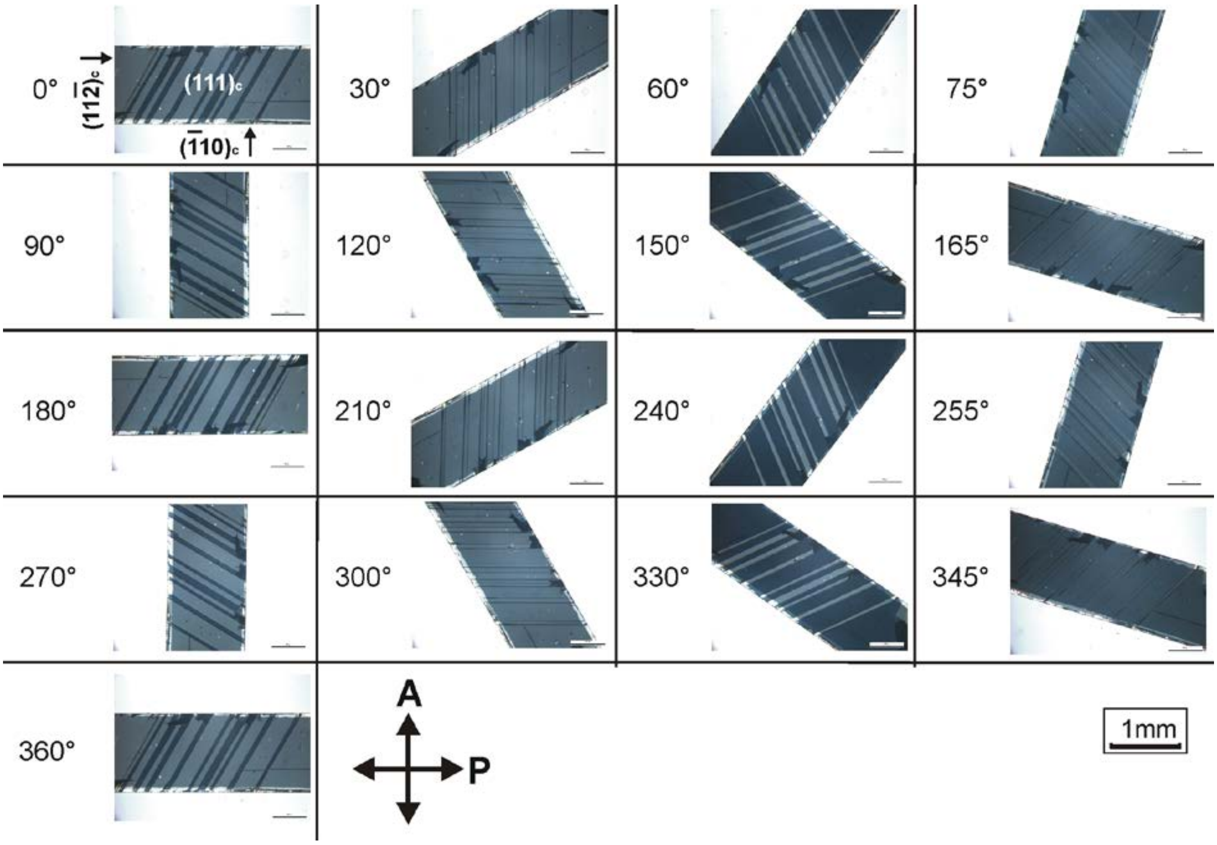

Supplementary Figure S3. Identification of the in-plane component of polarisation. 360-degree rotation of typical $(111)_c$ oriented $BaTiO_3$ crystal between crossed Nicols. Angles of rotation correspond to full darkening of one type of domains (0°, 90°, 180°, 270° and 60°, 150°, 240°, 330°) and equal intensity of transmitted polarised light through both of the domains (30°, 75°, 120°, 165°, 210°, 255°, 300°, 345°).

Polarisation analysis that we used in the present study assumes that the orientations of the polarisation vector and optical indicatrix in our samples are the same. To show this fact we plotted the intensity of propagated light through three different types of domains versus angle of analyser (Fig. 4S) for the fixed orientation of polariser. The orientation of polariser was set perpendicularly to the expected crystallographic orientation of polarisation. From these plots one can see that indeed the lowest transmission corresponds to perpendicular orientation of polariser and analyser when the orientation of optical indicatrix is parallel to the

analyser position. This allows us to make the conclusion that the optical indicatrix has the same orientation as the vector of polarisation.

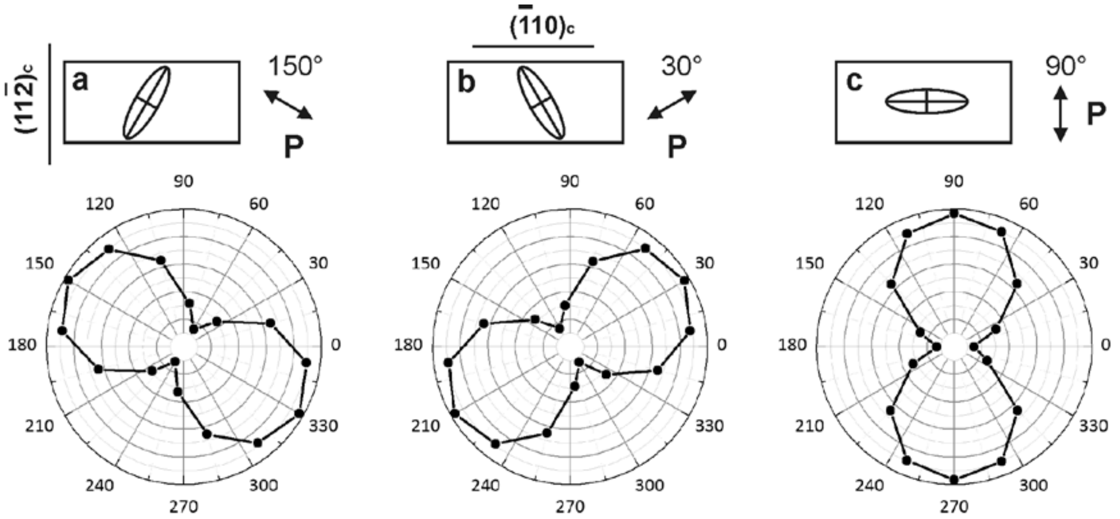

Supplementary Figure S4. Intensity of polarised light for different orientations of the analyser. 360-degree rotation of analyser for the fixed orientation of polariser (P) with typical $(111)_c$ oriented $BaTiO_3$ crystal in-between for three types of domains. The orientation of the polariser is set perpendicularly to crystallographic orientation of polarisation. Orientation of optical indicatrix corresponds to minimal intensity of light propagating through the domain and has the same orientation as the polarisation vector.

Supplementary Note 5: Domain pattern identification

To demonstrate the experimental approach for domain structure identification that we mainly used in the present work, we consider the example of the full identification of polarisation states in all domains (Fig. S5). The domain structure of a typical $(111)_c$ oriented $BaTiO_3$ crystal with charged domain walls is presented in three modes of optical microscope: reflected light (Fig. S5a), transmitted nonpolarised light (Fig. S5b) – intensity analysis, and transmitted polarised light (Fig. S5c) – polarisation analysis.

Figure S5a demonstrates the deformation of the surface due to ferroelastic 90-degree domains. The direction of the poling electric field used for sCDWs preparation is marked as an arrow pointing inward to the sample (plus on the top surface). The poling electric field was high enough to switch the out-of-plane polarisation of all domains into the direction of electric field (see Supplementary information Note 6). Further analysis takes into account that the out-of-plane component of polarisation in all domains is determined by the poling electric field and has the same out-of-plane direction. Domain walls in Fig. S5a have the $[0\bar{1}1]_c$ orientation which corresponds to charged ones because only sCDWs in the above described state have 60-degree angles to the $[11\bar{2}]_c$ edge in the $(111)_c$ plane (see Fig. 3).

Fig. S5b represents the same region of the domain pattern as Fig. S5b but in transmitted nonpolarised light. It shows the effect of refraction described in Fig. 4i called as intensity analysis, where one wall is brightened and another one is darkened. This picture allows us to identify which CDW is H-H and which is T-T. According to Fig. 4i brightened CDWs have H-H configuration and darkened CDWs have T-T configuration.

In Fig. S5c traditional observation in polarised ligh between crossed Nicols is presented – polarisation analyses. This method allows us to identify the orientation of the optical indicatrices in $(111)_c$ which are oriented parallel to the polarisation vectors. In this case (Fig. S5c) they are $[001]_c$ and $[010]_c$ (see Supplementary Information Note 4).

Thus the best way of CDWs identification in $(111)_c$ oriented crystals is simultaneous intensity and polarisation analysis having the polarisation direction predefined by the poling electric field.

For $BaTiO_3$ crystals of $(110)_c$ orientation, the combination of only two factors is sufficent for the domain structure identification: predefined polarisation direction by poling electric field together with intensity analysis.

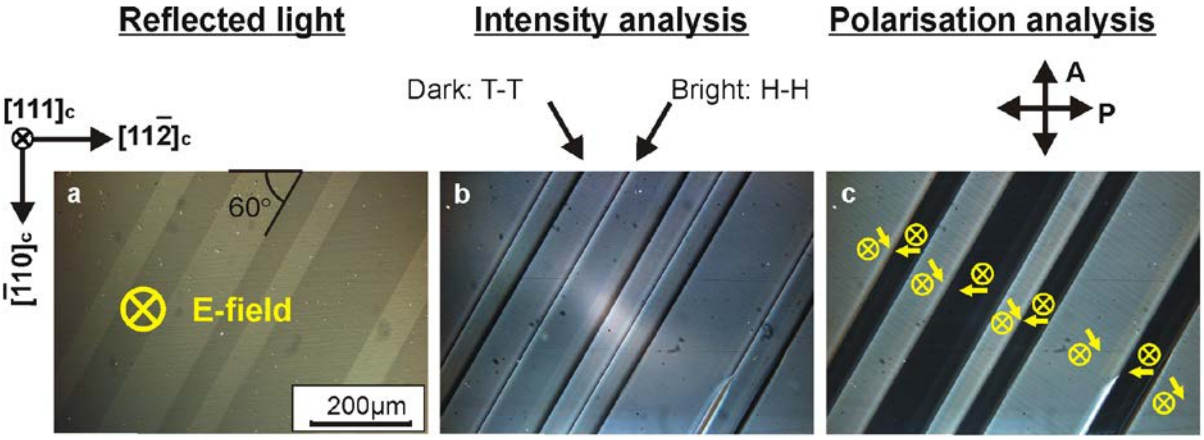

Supplementary Figure S5. Example of domain structure identification. Domain structure of typical $(111)_c$ oriented $BaTiO_3$ crystal with strongly charged domain walls observed in three modes of optical microscope: reflected light (a), transmitted nonpolarised light – intensity analysis (b) and transmitted polarised light – polarisation analysis (c).

Supplementary Note 6: sCDWs verification

For confirmation that the domain walls discussed in this work are charged, a simple method of verification is proposed (Fig. S6). $BaTiO_3$ crystal with already prepared presumably sCDWs (Fig. S6a) was poled in two opposite directions: along initial poling (Fig. S6b,c) and against initial poling (Fig. S6d-h). Poling electric field varied from values comparable to the coercive field 0.1kV/mm to values strongly exceeding it – 2kV/mm. It is clearly seen that poling along the direction of initial poling doesn't bring any significant changes in domain structure (Fig. S6b,c). On the contrary, poling against the direction of initial poling with electric field exceeding the coercive one, changes the contrast between domains (Fig. S6d-h). This is related to the rotation of polarisation vectors. With the red rectangle (Fig. S6b,e) is marked the moment when nothing changed in the first case while in the second case the structure is destroyed. It means that during opposite poling polarisation switched along the field while poling in the same direction as the initial one doesn't change the structure. Observed domain walls have the orientation of sCDWs (see Supplementary Information Note 5 and Fig. 3) that allows us to conclude that these walls are charged.

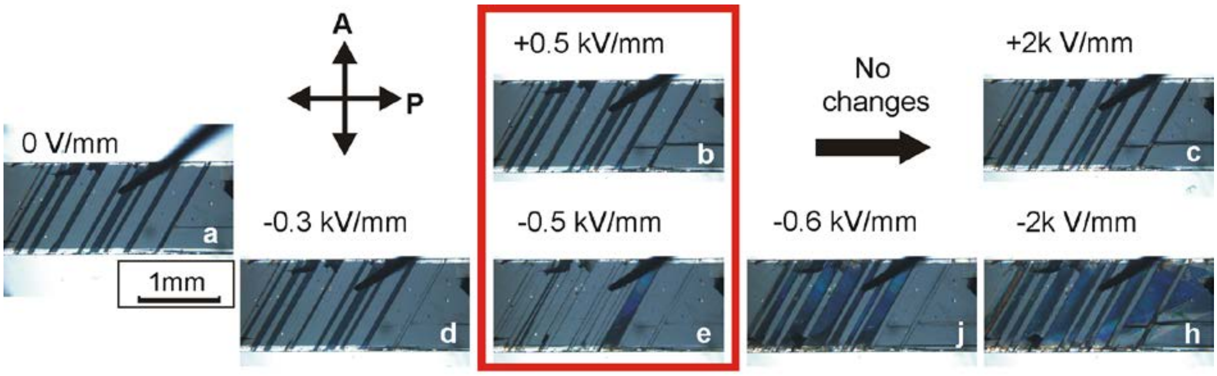

Supplementary Figure S6. Method of CDWs verification. Method of CDWs verification by means of poling in two opposite directions.

Note that poling against the direction of the initial field doesn't destroy CDWs themselves. Only rotation of polarisation according to electric field takes place. Change of contrast between domains (Fig. S6j-h) corresponds to a change of the optical indicatrix orientation. The rotation of the optical indicatrix observed in Fig. S6j-h appears due to the 90-degree switching of polarisation. Such switching is schematically demonstrated in Fig. S7. Thus, after poling in opposite directions darkened domains become light and light become darkened mostly keeping H-H and T-T sCDWs on their places.

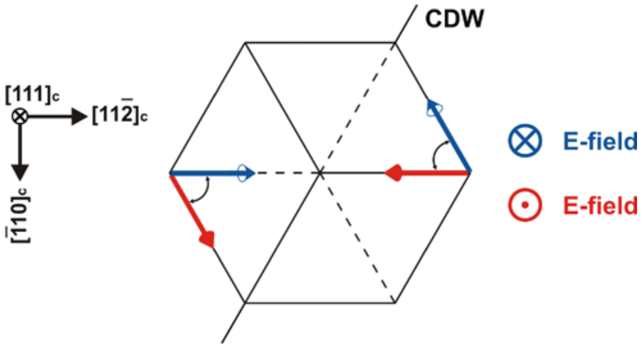

Supplementary Figure S7. Polarisation reversion. Polarisation vector rotation when poling in opposite directions: along $[111]_c$ and $[\bar{1}\bar{1}\bar{1}]_c$. Orientation of sCDW for both states: poled along $[111]_c$ and $[\bar{1}\bar{1}\bar{1}]_c$ directions is the same and shown schematically.

Supplementary Note 7: Conductivity of CDWs

To demonstrate the conductivity of CDWs we measured current through the sample with growing CDWs and without CDWs in the same crystal plate. Corresponding time dependence of integral current (current through the whole sample) and domain states are presented in Fig. S8.

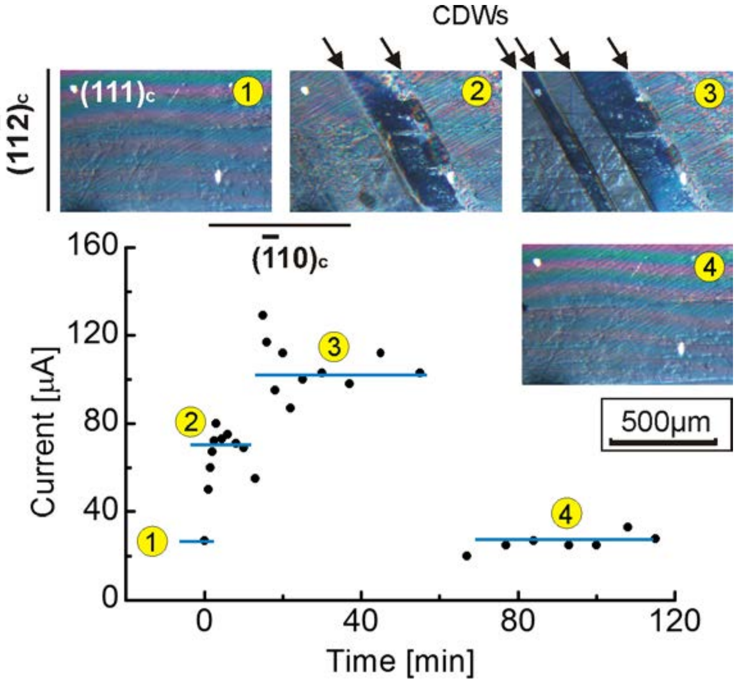

Supplementary Figure S8. Conductivity of CDWs. Time dependence of current through the sample S2 with and without CDWs: 1, 4 – without CDWs, 2 – with two CDWs, 3 – with four CDWs.

Supplementary Note 8: True conductivity estimation

For conductivity measurements one should take into account the influence of a low conductive interfacial layer. This layer appears due to the Schottky barrier in the region of metal-semiconductor contact. For the case of a Pt-$BaTiO_3$ contact, the Schottky barrier can be reduced by accumulation of oxygen vacancies [46]. After a short time of current stabilisation, while oxygen vacancies are not accumulated near the cathode, the main contribution to conductivity is related to the interfacial layer. After a long time of current stabilisation a high concentration of accumulated oxygen vacancies reduces the Schottky barrier and the conductivity of the bulk material can be measured.

We believe that following this scenario we can exclude the influence of the Schottky barrier. The observed current growth in all studied crystals during poling is a result of oxygen vacancy migration towards the cathode [41]. For this reasons the samples were kept under electric field more than 30 hours until the current was stabilised. Under the current stabilisation we assumed growth of current not more than 1 order of magnitude per 10 hours. Actually, the current continued growing even after 30 hours, but it was comparably small. This growth was taken into account by error bars described below. A high concentration of oxygen

vacancies near the electrode reduced the Schottky barrier and allowed measurement of the true conductivity of the material.

For experimentally measured conductivities (samples S2, S4, S5) error bars are taken as experimentally measured value (lower boundary) plus one order of magnitude for S2, S4 and two orders of magnitude for S5. For S5 sample error bar is longer because the current exceeded the operational limits of the measuring device and after a certain value of current it was not possible to perform measurements. In the present study the upper boundary is not as important as the lower one. To satisfy the electron/ion screening scenario the free charge density should be above the theoretical curve plotted in Fig. 5 that requires the lower boundary to be close to the theoretical curve.

To characterise artificially oxygen depleted crystals S1, S3, S5, their conductivities were estimated from the literature data [39-40]. In Fig. S9 the dependence of conductivity of quenched samples vs. oxygen partial pressure in the furnace during annealing is presented. For the sample S1 the conductivity could not be measured correctly because an interfacial low conductive layer could not be reduced by oxygen vacancies due to a low oxygen deficiency in the crystal. Only the conductivity of the barrier could be measured. Thus, conductivity of sample S1 was estimated from Fig. S9.

From Fig. S9 one can see that in crystals annealed at 800°C and 1000°C significant conductivity growth takes place at different oxygen partial pressures. Similar high conductivity of $BaTiO_3$ crystals was experimentally observed (e.g. experimental data for S5 marked with green empty dot in Fig. S9) for annealing at 1200°C when oxygen partial pressure is 10^{-8} atm. This fact gives us information that strong conductivity growth in $BaTiO_3$ crystals annealed at 1200°C appears at a pressure of 10^{-8} atm or even higher.

For the sample S3 measurements were not performed because the sample was destroyed during the experiment. Sample S3 was annealed close to the oxygen pressure corresponding to conductivity growth demonstrated experimentally for S5 sample. The error bar for S3 is taken in a wide conductivity range between S5 and S1 samples.

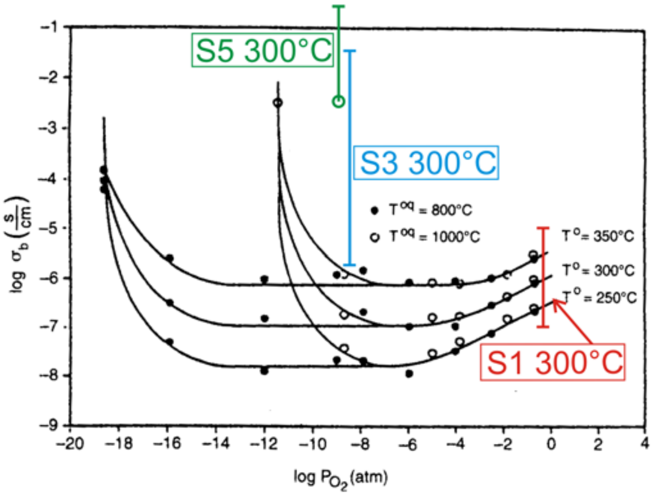

Supplementary Figure S9. Estimation of conductivity. The conductivities of $BaTiO_3$ crystals measured for samples quenched from 800 and 1000°C and measured at 250, 300 and 350°C. Conductivity estimation from literature data for artifitially oxygen depleated crystals [39-40]. Coloured markers show the estimated conductivity of S1, S3, S5 samples.

Concentration of oxygen vacancies in S1-S5 crystals was estimated from their conductivity and is presented in table S1.

**Sample**	**Conductvity [S/m]**	**Concentration of Vö [m^{-3}]**
**S1**	10^{-5}-10^{-3}	10^{18}-10^{20}
**S2**	10^{-2}-10^{-1}	10^{21}-10^{22}
**S3**	10^{-3}-10^{1}	10^{20}-10^{24}
**S4**	10^{-1}-10^{0}	10^{22}-10^{23}
**S5**	10^{0}-10^{2}	10^{23}-10^{25}

Supplementary Table S1. Characterisation of the samples. Studied types of $BaTiO_3$ crystals with their conductivity obtained at 300°C, and concentration of oxygen vacancies evaluated from the conductivity.

43. Iwata, M. and Ishibashi, Y. Theory of morphotropic phase boundary in solid solution systems of perovskite-type oxide ferroelectrics: p-e hysteresis loop. *Jpn. J. Appl. Phys. Part 1-Regular Papers Short Notes & Review Papers*. **38**(9B), 5670-5673 (1999).
44. Lemanov, V.V., Smirnova, E. P., Syrnikov, P.P., Tarakanov, E. A., Phase transitions and glasslike behavior in $Sr_{1-x}Ba_xTiO_3$. *Phys. Rev. B*. **54**(5), 3151-3157 (1996).
45. Bell, A.J., Phenomenologically derived electric field-temperature phase diagrams and piezoelectric coefficients for single crystal barium titanate under fields along different axes. *J. Appl. Phys.* **89**, 3907 (2001).
46. Zhang, T.J. et al. Large rectifying leakage current in Pt/$BaTiO_3$/Nb:$SrTiO_3$/Pt structure. *Appl. Phys. Lett.* **99**, 182106 (2011).
